# Viral reservoir seeding and neurological metabolic dysregulation in early-life immunodeficiency virus infection

**DOI:** 10.21203/rs.3.rs-7802364/v1

**Published:** 2025-10-12

**Authors:** Li Ma, Robert Blair, Yao-Zhong Liu, Xiaolei Wang, Eunice Vincent, Christopher Yu, Lara A Doyle-Meyers, Cissy Zhang, Ahmad Saied, Anne Le, Ronald S Veazey, Qigui Yu, Huanbin Xu

**Affiliations:** Tulane National Primate Research Center (TNPRC); Tulane National Primate Research Center (TNPRC); Tulane University; Tulane National Primate Research Center (TNPRC); Tulane National Primate Research Center (TNPRC); Indiana University School of Medicine; Tulane National Primate Research Center (TNPRC); Gigantest; Tulane National Primate Research Center (TNPRC); Gigantest; Tulane National Primate Research Center (TNPRC); Indiana University School of Medicine; Tulane National Primate Research Center (TNPRC)

**Keywords:** HIV/SIV, infants, viral reservoirs, brain pathology, metabolic alterations, early treatment

## Abstract

**Background:**

Viral reservoirs are rapidly established in peripheral and lymphoid tissues in HIV-exposed and infected infants, but the timing and consequence of viral seeding in the brain tissues remain unclear.

**Methods:**

Based on previously collected samples, this study retrospectively examined early viral reservoir seeding in the brain, neuropathological alterations, and cerebrospinal fluid (CSF) metabolic changes in neonatal rhesus macaques infected with SIV at birth, with or without early initiation of antiretroviral therapy (ART).

**Results:**

Viral RNA was detectable in CSF by 2 days post infection (dpi), paralleling plasma viral load, whereas brain tissue-associated viral DNA and RNA were not consistently detected until after 3 dpi. No significant neuropathological lesions or CSF proinflammatory responses were observed during the first week of infection. In contrast, profound CSF metabolic dysregulation emerged, which was largely reversed by early ART, particularly when initiated at 3 dpi.

**Conclusions:**

These findings define the kinetics of brain viral reservoir seeding in neonates and highlight the critical importance of initiating ART within the earliest stages of infection to preserve brain development and function in children exposed to maternal HIV.

## Background

Despite the recommendations for maternal antiretroviral therapy (ART), cesarean delivery, and formula feeding to significantly reduce vertical transmission rates from 25% to less than 2%, HIV acquisition in children continues to occur. Globally, approximately 150,000 infants and children are infected with HIV annually (UNAIDS, 2022) [1]. These infants exhibit rapid viral reservoir seeding and disease progression, with a high risk of viral rebound if treatment is discontinued or interrupted [2–12]. Our recent study indicates that both plasma viral load and SIV RNA/DNA in systemic and lymphoid tissues of neonatal macaques infected with SIV within 24 hours of birth are readily detectable as early as 1 day post inoculation (dpi). Further, a 9-month daily combined ART regimen (FTC/TFV/DTG, cART), initiated at 3 dpi, resulted in ART-free viral remission in 80% of infant [13]. These findings suggest that sustained virological remission is achievable through early ART treatment in pediatric patients.

HIV/SIV can enter the central nervous system (CNS) through the “Trojan horse” mechanism, crossing the blood-brain barrier [14–16]. As a distinct anatomical compartment, the CNS serves as a sanctuary site for HIV reservoirs even after ART initiation, owing to suboptimal penetration of antiretrovirals (ARVs) and the long lifespan of infected macrophages and astrocytes. In adults, viral reservoirs in the brain have been detected as early as 1–2dpi [17–20]. Given the difference in early viral reservoir establishment in systemic and lymphoid tissues of infants compared with adults, it remains unclear whether the dynamics of early brain reservoirs seeding in neonates also differ from those in other tissues or in adults. Neonatal macaques share similarities with human infants, including brain development patterns and immune system maturation [21, 22]. Defining the early events of brain viral reservoirs is critical for optimizing pediatric ART timing to prevent viral reservoir establishment and encephalitis, thereby supporting heathy brain development and function in children.

In this study, we examined the dynamics of early brain-associated viral reservoir seeding in neonatal macaques infected with SIV at birth, along with associated neuropathological changes and inflammatory responses. We also compared cerebrospinal fluid (CSF) metabolites in one-month-old infants, focusing on cohorts in which ART was initiated at 3, 4, or 5 dpi, as a two-day treatment delay fails to achieve viral remission after analytical treatment interruption (ATI) [23]. Our results demonstrated that both viral DNA and RNA were undetectable in brain tissue until after 3 dpi, a pattern distinct from that observed in peripheral tissues of neonatal or adult macaques. Further, there were no significant neuropathological changes or CSF proinflammatory responses in neonates during the first week of infection. Importantly, early ART largely normalized CSF profiles in SIV-infected neonatal macaques. Together, these findings highlight the critical importance of timely ART initiation to preserve normal brain development and function in HIV-exposed and infected infants.

## Methods

### Animals and samples

A total of 35 Indian-origin rhesus macaques (*Macaca mulatta*), with a random sex distribution, were included in this study. Four animals served as SIV-naïve controls. The other animals, newborn macaques (n = 25) and juveniles (n = 6), were intravenously infected with 100 TCID50 SIV. Among the SIV-infected newborns, animals were euthanized at 1dpi (n = 2), 2dpi (n = 3), 3 dpi (n = 6), 5 dpi (n = 2), and 7dpi (n = 3). The remaining nine neonatal animals received a one-month course of early combined antiretroviral therapy (cART; FTC/TFV/DTG) as previously described, initiated at either 3 dpi (n = 3), 4 dpi (n = 3), or 5 dpi (n = 3). An additional group of three age-matched, untreated SIV-infected neonates served as infection controls. Plasma, cerebrospinal fluid (CSF), and brain tissues collected from these animals in prior studies [13, 24] were retrospectively analyzed for early brain viral reservoir seeding, neuropathology, and CSF metabolic alterations in the present study.

### Nucleic acid extraction of plasma, CSF and brain tissues

Brain tissues (Frontal cortex, Hippocampus and Basal ganglia) were processed for extraction of genomic DNA and total RNA using the AllPrep DNA/RNA Mini Kit (Cat No: 80311, Qiagen). In brief, ~ 50 mg of tissue was homogenized in 700 μL Buffer RLT Plus supplemented with 80 μL Proteinase K solution (20 mg/mL, Invitrogen, Cat. No. 2935092), followed by DNA and RNA isolation according to the manufacturer’s instructions. Viral RNA from plasma and CSF was extracted using the QIAamp Viral RNA Mini Kit (Qiagen, Cat. No. 52962). All DNA and RNA samples were stored at − 80°C until use.

### Quantification of viral load and brain tissue-associated SIV DNA/RNA

Specific primer and probe sets targeting the SIV *gag* region [13, 23], spliced SIV *tat*, and rhesus RPPR30 were synthesized by integrated DNA technologies. The sequences were as follows: SIV gag: Forward GTC TGC GTC ATC TGG TGC ATT C; Reverse CAC TAG GTG TCT CTG CAC TAT CTG TTT TG; and probe FAM-CTT CCT CAG TGT GTT TCA CTT TCT CTT CTG CG-BHQ-1). Spliced SIV tat: Forward GAA CTC CGA AAA AGG CTA AGG CTA ATA CA; Reverse CCG TCT CCT TCT TTG CCT TCT CTG GTT; and probe ABY-CTG CAT CAA ACA ACA GAC CCA TAT CCA ACA GGA CC-QSY. Rhesus RPPR30: Forward TCA GCA TGG CGG TGT TT; Reverse GCT GTC TCC ACA AGT C; and probe VIC-TTC TGA CCT GAA GGC TCT GCG C-3IABkFQ-1). The SIV gag primer/probe set was used to determine viral load in plasma and CSF (LOD, 30copies/mL). Viral RNA from plasma and CSF was reverse transcribed into cDNA using the ThermoFisher SuperScript^™^ VILO^™^ Master Mix (Invitrogen, Grand Island, NY). Reactions were performed on a thermocycler at 25°C for 10 min, a 42°C for 60 min, and enzyme inactivation at 85°C for 5 min. cDNA was then used to quantify SIV transcripts by digital PCR on the QuantStudio Absolute Q Digital PCR System (Applied Biosystems, Thermo Fisher, US) using the Absolute Q^™^ MAP16 Plate Kit and Master Mix. Cycling conditions were: 96°C for 10 min, followed by 40 cycles of 96°C for 15s and 60°C for 30s. For brain tissue samples, cellular input was normalized by quantifying RPPR30 genomic copies (two copies per rhesus macaque cell). Quantification of SIV DNA/RNA were performed using a minimal of 2×10^5^ cell number equivalents per sample, and results were expressed as copies per one million cells (LOD, 5 copies/mL). For samples with undetectable or very low SIV copy numbers, nested quantitative digital PCR, capable of detecting single-copy target, were further performed to confirm true absence or accurately quantify low-level copies.

### Cytokine assay

Proinflammatory cytokines in plasma and CSF were measured by Luminex 200 systems (Bio-Rad Inc., Hercules, CA, USA) according to the manufacturer’s instructions, as we previously described [24, 25]. Cytokine levels were measured using the ProcartaPlex NHP cytokine/GF37plex (Invitrogen). Briefly, 50 μl diluted plasma (1∶1) was incubated with antibody-coupled beads. Complexes were washed, incubated with biotinylated detection antibody, and then streptavidin-PE. Cytokine levels were determined using a multiplex array reader from Luminex^™^ Instrumentation System (Bio-Plex Workstation from Bio-Rad Laboratories). Analyte concentrations were calculated using software provided by the manufacturer (Bio-Plex Manager Software).

### Brain tissue pathological assessment

Brain tissue sections were collected from neonatal macaques euthanized at 1–7dpi following SIV infection. Gross pathological observations included the presence, location, size, number, and distribution of lesions. Samples were fixed in buffered 10% formalin with ionized zinc (Z-Fix^™^, Anatech LTD, Battle Creek, MI), embedded in paraffin, and sectioned at 5 μm for routine hematoxylin and eosin (H&E) staining. Pathological evaluation was performed by a board-certified veterinary pathologist (ACVP-certified) on multiple brain regions (frontal cortex, temporal cortex, parietal cortex, occipital cortex, basal ganglia, thalamus, hippocampus, cerebellum, and brainstem) and included assessment for each of the following lesions: cortical and perivascular mineralization, hemorrhage, ependymal loss, and microhemorrhage (no other lesions were noted across the cohort). Each lesion was scored in each section independently as follows: 0 (absent), 1 (minimal), 2 (mild), 3 (moderate), and 4 (severe). Cumulative pathology scores were generated for each animal by summating the lesions scores across all sections for an animal resulting in the following scoring scale: 0 (absent), 1–5 (minimal), 6–10 (mild), 11–15 (moderate), and > 15 (severe), reflecting the overall severity of brain pathology. Digital slides were viewed and imaged using HALO v3.6 (Indica Labs).

### Metabolomics analysis of cerebrospinal fluid samples

Cerebrospinal fluid (CSF) was collected by cisterna magna puncture under aseptic conditions, immediately placed on ice, and centrifuged at 2,000×g for 10 min at 4°C to remove cells. Supernatant aliquots were stored at − 80°C until analysis. As previously described [26, 27], for metabolite extraction, 100 μL of cell-free CSF was transferred to a 1.5-mL centrifuge tube and mixed with 400 μL cold extraction solvent (acetonitrile: methanol,1:1 v/v) containing an internal standard mixture (stable-isotope labeled standards; final concentration 0.02 mg/mL for each standard) was added. Samples were vortexed for 30 s and solicited in an ice-cooled bath (≈ 5°C, 40 kHz) for 30 min. Proteins were precipitated by incubation at − 20°C for 30 min, followed by centrifugation at 13000 × g for 15 min at 4°C. The supernatant was collected and evaporated to dryness under a gentle stream of nitrogen. Dried extracts were reconstituted in 100 μL of acetonitrile:water (1:1, v/v), sonicated for 5 min at low temperature, and centrifuged at 13,000×g for 10 min at 4°C. Final supernatants were transferred to LC-MS/MS vials (optionally passed through a 0.2 μm centrifugal filter) for analysis. A pooled quality control (QC) sample was prepared by combining equal aliquots of all study samples and processed alongside study samples. Chromatographic separation was performed on column (Waters Acquity BEH C18, 2.1 × 100 mm, 1.7 μm) at 40°C with a flow rate of 300 μL/min. Mobile phases were (A) water + 0.1% formic acid (v/v) and (B) acetonitrile + 0.1% formic acid (v/v). Injection volume was 3 μL and the total run time was 10 min. Mass spectrometric detection was performed on a Q Exactive^™^ Plus Hybrid Quadrupole-Orbitrap^™^ Mass Spectrometer with Vanquish Duo UHPC systems (ThermoFisher Scientific). Raw data were pre-processed using Progenesis QI software (Waters Corporation, Milford, USA) for alignment, peak picking, and initial identifications and then further analyzed in R (Version 4.2.2) with Bioconductor packages. Metabolite identities were confirmed where possible against authentic standards or spectral libraries. Statistical testing used Benjamini–Hochberg FDR correction (FDR < 0.05 considered significant). Visualization of significant metabolites was done via heatmaps using the pheatmap R package. Intensities were z-score normalized, and hierarchical clustering was applied (both samples and metabolites). To explore relationships between metabolite classes, individual metabolites, and metabolic pathways (for upregulated vs. downregulated metabolites), Sankey diagrams were created using the networkD3 package.

### Statistical analysis

Statistical analyses were performed by non-parametric Mann-Whitney t test using GraphPad Prism 10.3 Software (GraphPad Software, San Diego, CA). A nominal α level of 0.05 was used to define statistical significance and the data are presented as mean and SEM.

## Results

### Delayed establishment of brain viral reservoirs in neonatal macaques following SIV infection at birth

**Figure 1:**
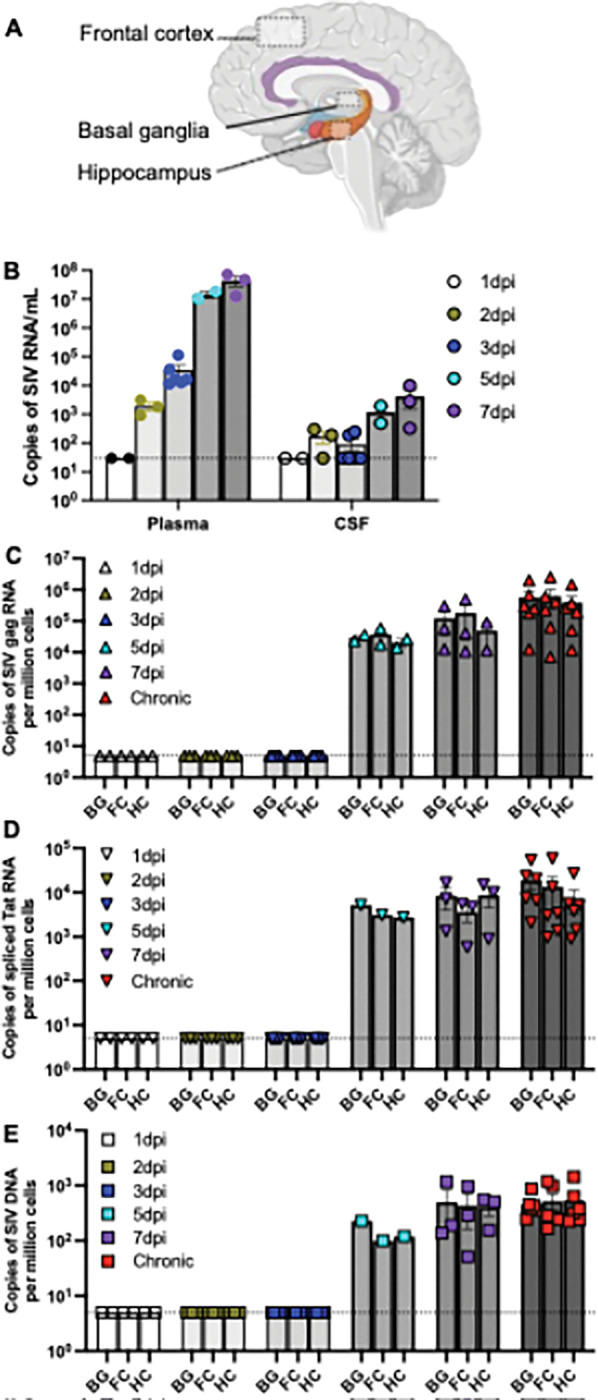
Early brain viral reservoir seeding in neonatal macaques infected with SIV within 24 hours of birth. (**A**) Schematic representation of brain tissue collection sites for brain viral reservoir detection in SIV-infected neonatal macaques. (**B**) Viral loads in plasma and cerebrospinal fluid (CSF) samples collected during the first week post infection; (**C-E**) Levels of cellular SIV *gag* RNA (C), spliced SIV *tat*RNA (D), and SIV DNA (E) in the basal ganglia (BG), frontal cortex (FC), and hippocampus (HC) of neonatal macaques during the first week post infection, compared with brain tissues section from chronically SIV-infected juveniles. Note that spliced SIV *tat* RNA represents actively transcribed viral RNA, rather than residual inoculum. CSF viral load was detectable as early as 2dpi, whereas brain viral reservoirs were not consistently detected until after 3 dpi.

In SIV exposed and infected newborn macaques, rapid viral replication was evident, with plasma viral load detectable in two of four animals at 1dpi and in all neonates at 2dpi. In contrast, viral load in cerebrospinal fluid (CSF) displayed a delayed pattern, remaining undetectable in some animals at 3 dpi (Figs. 1A–1B). Unexpectedly, cell-associated SIV *gag* RNA was absent in representative basal ganglia (BG), frontal cortex (FC), and hippocampus (HC) tissue sections within the first 3 dpi but became detectable in these tissues at 5 dpi and thereafter (Fig. 1B). This pattern was fully consistent with the detection of spliced SIV *tat* RNA and SIV DNA in brain tissues, which also appeared from 5 dpi through the chronic stage (Fig. 1C–1D). Notably, spliced SIV *tat* RNA reflects active viral replication rather than contamination from virus inoculum or circulating virions [28, 29]. Collectively, these findings indicate that while rapid viral replication and dissemination occur in systemic and lymphoid tissue compartments of neonates [13], viral reservoirs are not established in the brain within the first 3 days post infection following SIV exposure at birth. This temporal delay, distinct from reservoir seeding in systemic and lymphoid tissues, provides a critical window in which timely pediatric ART could prevent the establishment of brain viral reservoirs, thereby minimizing the long-term impact of brain HIV reservoir on brain development and function in growing children.

### Limited proinflammatory responses in neonatal macaques infected with SIV at birth

**Figure 2:**
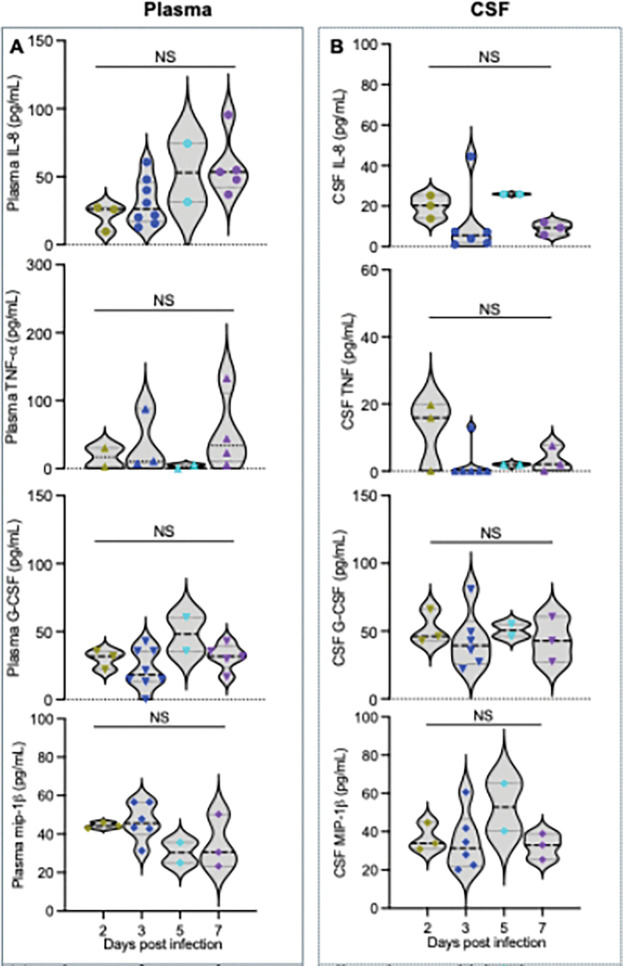
Proinflammatory mediator dynamics in plasma and CSF of neonatal macaques during the first week post SIV infection. (**A-B**) Changes of representative proinflammatory cytokines and chemokines, including IL-8, TNF-a, G-CSF, and MIP-1β, measured in plasma (A) and CSF (B) of neonatal macaques during the first week after SIV infection. NS indicates no significant difference (p > 0.05).

We previously reported that plasma cytokine levels do not significantly increase during acute SIV infection in infants compared with adults [24, 25]. To further characterize proinflammatory responses in the neonatal CSF, particularly during the first week following SIV infection, we comparatively measured proinflammatory cytokine and chemokine levels in both plasma and CSF samples. As shown in Figs. 2A and 2B, these typical proinflammatory mediators, including IL-8, TNF-α, G-CSF and MIP-1β, did not differ significantly across time points or between plasma and CSF post infection, consistent with findings for other cytokines and chemokines included in the kit. Taken together, these results indicate that very early proinflammatory responses in both plasma and CSF are limited in neonatal macaques infected with SIV at birth, which may mitigate innate immune activation, meningitis, and potential brain pathology.

### Minimal brain pathological changes in neonatal macaques during early SIV infection

**Figure 3:**
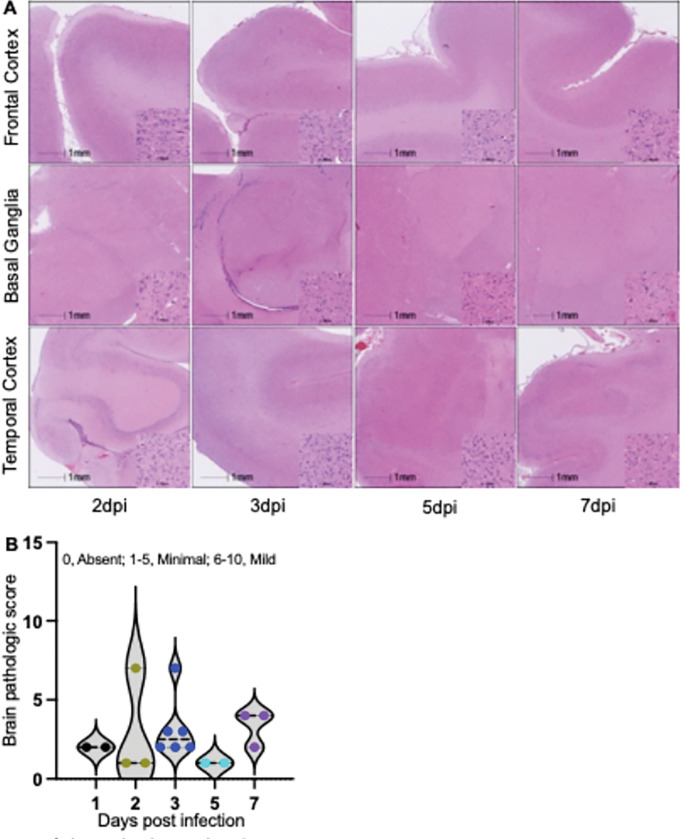
Histopathological assessment of brain tissues in neonatal macaques during the first week of SIV infection. (**A**) Representative H&E-stained sections of the frontal cortex, basal ganglia, and temporal cortex from SIV-infected neonatal animals collected at 2, 3, 5, and 7dpi. Scale bar = 1 mm. Note that significant pathology is not observed in brain regions examined during the first week of SIV infection. (**B**) Aggregate histopathologic scores across multiple brain regions, including frontal cortex, temporal cortex, occipital cortex, basal ganglia, thalamus, hippocampus, cerebellum, and brainstem, during the first week post SIV infection. Cumulative score system: 0, absent; 1–5, minimal; 6–10, mild; 11–15, moderate; >16, severe.

In parallel with assessing the dynamics of brain viral reservoir establishment in neonatal macaques during the first week following SIV infection, we comprehensively evaluated neuropathologic changes. Brain tissue sections, including, but not limited to, the frontal cortex, basal ganglia, and temporal cortex, were examined and independently scored for each of the following lesions: hemorrhage, infarction, cortical and perivascular mineralization, ependymal loss, inflammation, and degeneration. As shown by H&E staining (Fig. 3A), no significant inflammatory lesions were detected in the brain sections within 7dpi, nor were there pathologies directly attributable to SIV infection. Minimal and scattered findings, such as focal cortical or perivascular mineralization, segmental ependymal cell loss, and microhemorrhages, were observed, but there was no evidence of degeneration, apoptosis, or necrosis. A standardized cumulative pathology score for each neonate, across all examined sections (0, absent; 1–5, minimal; 6–10, mild; 11–15, moderate; >16, severe), revealed minimal changes in 14 of 16 neonates and mild changes in 2 or 16 neonates with no pathology (inflammation, degeneration, apoptosis, or necrosis) during the first week of infection (Fig. 3B). These findings indicate that brain pathology in neonates is scarce during very early SIV infection, consistent with the limited proinflammatory responses in CSF.

### Reversal of CSF metabolic dysregulation induced by neonatal SIV infection through timely early antiretroviral therapy

**Figure 4:**
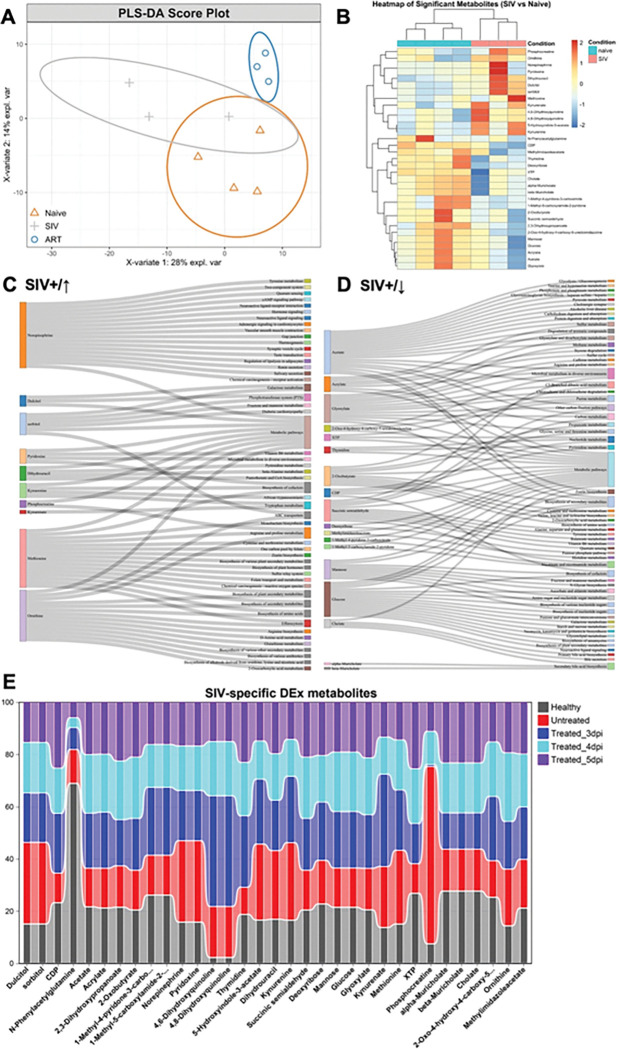
CSF metabolic alterations in neonatal macaques infected with SIV within 24 hours of birth, with or without early treatment initiation. (**A**) PLS-DA score plot showing distinct metabolic clustering across experimental animal groups. (**B**) Heatmap of significantly altered SIV-specific metabolites in CSF from untreated SIV-infected neonates, compared with SIV naïve controls. Rows represent metabolites, columns represent individual samples. Z-score indicate relative metabolite abundance (scale bar: red, upregulated; blue, downregulated). (**C-D**) Sankey diagrams depicting SIV-specific upregulated (C) and downregulated (D) metabolites and their associated biological pathways/functions. (**E**) Effect of early treatments on CSF metabolites, compared with both SIV naïve and untreated SIV-infected neonates. A total of 34 SIV-associated differentially regulated metabolites were identified and present here, most of which were restored by early treatment, particularly when initiated at 3 dpi. Animal groups included SIV naïve controls (n=4), untreated SIV infected neonates (n=3), and SIV infected neonates treated with one-month of cART starting at 3 dpi (n=3), 4dpi (n=3), or 5 dpi (n=3). Newborn macaques (n=12) were intravenously infected with an identical SIV inoculum, with CSF samples collected at 1 month post SIV infection. The data are shown as p-adjusted metabolite intensity normalized at percentage scale across 5 cohorts.

Our previous studies demonstrated that pediatric ART initiated at 3 dpi can achieve sustained virologic remission in most infants following discontinuation of a 9-month treatment regimen [13]. In contrast, initiation at 5 dpi, a delay of just two days, resulted in viral rebound after ART interruption [23]. To assess the impact of neonatal SIV infection and early ART on brain-associated metabolism, we analyzed CSF samples collected from one-month-old infant cohorts, including SIV-naïve (healthy) controls, untreated SIV-infected animals, and SIV-infected animals with ART initiated at 3, 4, or 5 dpi. Based on overall metabolic patterns, partial least squares–discriminant analysis (PLS-DA) revealed a clear separation between SIV-naïve and untreated SIV-infected groups along the first major component (i.e., x-variate 1) that explained ~ 30% of data variability. In contrast, only minor differences metabolic differences were observed between SIV-naïve and SIV-infected groups that received early cART beginning at 3 dpi along the first major component, suggesting that neonatal SIV infection induces a profound systemic shift in the CSF metabolome that can be reversed toward a homeostatic state by early cART. Variation along the 2nd major component (x-variate 2) are likely due to individual animal differences and ARV-specific effects rather than primary SIV infection (Fig. 4A). Analysis at levels of individual CSF metabolites between SIV-naïve and untreated SIV-infected groups showed distinct alterations in CSF metabolites. The heatmap displayed a clear enrichment of a list of in the untreated SIV-infected group, including phosphocreatine, sorbitol/dulcitol, kynurenine, norepinephrine, ornithine, and dihydrouracil (Fig. 4B). These changes, consistent with the PLS-DA results, reflect severe energy metabolism dysregulation, oxidative stress and neuroimmune suppression, and increased cellular turnover, as a signature of pathological processes. Conversely, other metabolites were depleted or downregulated in the SIV-infected group, including bile acids, key energy substrates (glucose, mannose and acetate), and nucleotides (XTP, CDP, deoxyribose and thymidine) (Fig. 4B), suggesting metabolic disruption for protective factors and energy sources essential for maintaining CNS environment during neonatal SIV infection. As shown in Figs. 4C-4D, the Sankey plots provided a mechanistic overview linking altered metabolites to their associated biological pathways and functions, highlighting the metabolic encephalopathy induced by neonatal SIV infection. For example, infection drove hyperactivation of dysregulated galactose metabolism (sorbitol/dulcitol), tryptophan and microbial metabolism (kynurenine/kynurenate), and neuronal signaling pathways (phosphocreatine and norepinephrine) (Fig. 4C). In contrast, depletion of key metabolites revealed compromised pathways, including bioenergetic deficits (glucose, mannose, acetate), impaired biosynthesis and secretion (bile acids), collapse of nucleotide synthesis and turnover, and damage of systemic processes (e.g., glyoxylate, succinic semialdehyde) (Fig. 4D). These findings suggest that neonatal SIV infection induces a catabolic state marked by energy failure, loss of neuroprotection, and broad suppression of biosynthetic processes. Finally, when early treatment was initiated in neonatal macaques, the trajectories of each metabolite significantly altered in untreated SIV-infected animals were tracked. Early ART reversed pathological metabolic profiles in a time-dependent manner. For example, metabolites that were elevated in untreated animals (phosphocreatine, sorbitol/dulcitol, kynurenine) were reduced, while those that were depleted (bile acids, glucose/mannose, XTP) were restored following treatment, most prominently when initiated at 3 dpi, though not fully normalized by 5 dpi. Notably, treatment at 3 dpi regulated the most pronounced metabolic shift in CSF, moving the profile closer to that of healthy controls compared with treatment at 4 or 5 dpi (Fig. 4E). Collectively, these findings demonstrate that neonatal SIV infection induces significant metabolic disturbances in the CSF, while timely early ART can substantially reverse this imbalance, potentially replenishing energy substrates and neuroprotective compounds essential for CNS homeostasis.

## Discussion

Children exposed to and infected with maternal HIV frequently experience neurodevelopmental delays, cognitive impairment, and mental health disorders [30–33]. Yet, the impact of neonatal HIV infection at delivery on early brain reservoir seeding, encephalopathy, and cerebrospinal fluid (CSF) metabolic alterations, particularly in untreated versus early-treated infants, remains poorly understood, largely due to ethical and practical limitations in obtaining brain samples. Utilizing a neonatal macaque model of SIV infection, we investigated viral reservoir establishment and neuropathological changes during the first week of infection and evaluated CSF metabolic profiles at one month of age in the presence or absence of early ART. Our findings show that brain reservoirs were not consistently established until after 3 dpi, that neuropathology and neuroinflammation were minimal during this window, and that SIV infection caused profound CSF metabolic dysregulation that was largely restored by early treatment. These findings highlight the importance of initiating ART at the earliest therapeutic opportunity to preserve brain development and mental health in children living with HIV.

Understanding reservoir establishment in the neonatal CNS following maternal HIV infection is critical for developing strategies to mitigate neurological complications and optimize early prevention interventions. In adults, viral reservoirs are found in microglia, macrophages, astrocytes, and T cells across multiple brain regions [34–40], where intact proviruses persist despite long-term ART [22, 41, 42]. These reservoirs are seeded when infected cells migrate across the blood–brain barrier (BBB) [14, 20, 43, 44]. Our previous study demonstrated that viral DNA, but not integrated proviral DNA, could be detected in systemic and lymphoid tissues of neonates as early as 1dpi [13]. Here we showed that while SIV RNA was detectable in CSF by 2dpi, consistent with earlier infant macaque study [45], both SIV DNA and RNA were absent from brain tissues until after 3 dpi, as confirmed by digital PCR and nested digital PCR. Spliced SIV tat transcripts in the brain paralleled SIV gag RNA levels, reflecting active replication from local viral DNA rather than residual inoculum. These findings help explain why initiating ART at 3 dpi in neonates can eliminate early viral reservoirs and achieve sustained virologic remission, before the establishment of brain reservoirs that represent major barriers to cure [13]. In other studies were neonatal macaques were either infected with SIV within 24 hours, 1 or 3 days after birth, SIV DNA/RNA in brain tissues remains essentially undetectable but could be detected by 7dpi, accompanied by histopathological lesions at 14dpi and progressive reductions in brain growth thereafter [46–48]. In contrast, older infant macaques (3 weeks-5 months) exhibit earlier brain reservoir seeding, with SIV DNA detectable as early as 1–2dpi [19, 47, 49]. Despite detectable systemic viral dissemination, neonatal SIV infection during the first week caused only limited neuropathological changes. We observed no significant inflammatory lesions or degeneration in brain tissue sections and only minimal scattered changes (e.g., mineralization, microhemorrhages, segmental ependymal loss). Consistently, both plasma and CSF cytokine analyses showed limited proinflammatory responses, echoing prior reports of limited cytokine induction in infant macaques [24]. This relative lack of early neuroinflammation may delay the onset of neuropathology compared to older animals. Interestingly, hippocampal neuronal loss has been reported to be more pronounced in neonates infected orally compared to intravenously [50], suggesting the contribution of infection route to neuropathogenesis. Together, these discrepancies in the timing of viral seeding and neuropathology between neonates and older infants are likely attributable to differences in age, blood–brain barrier permeability, abundance of brain target cells, and inoculation route [22, 46, 48, 50–52].

Although early neuropathological changes were limited, metabolomic profiling revealed profound CSF metabolic disturbances at one-month post SIV infection. CSF metabolites are tightly linked to neurological development, cognition, and brain physiology [53–56]. Untreated neonates exhibited a multimodal metabolic crisis marked by energy substrate depletion (glucose, mannose, acetate, succinic semialdehyde), increased oxidative stress, and dysregulated neurotransmitter metabolism. Elevated kynurenine, norepinephrine, 5-HIAA, and sorbitol/dulcitol suggest neuroinflammation, immune suppression, oxidative stress, and neurotoxicity, while reduced bile acids point to the loss of neuroprotective signaling [57–60]. Together, these alterations reflect a vulnerable neurochemical state that may predispose to long-term developmental impairments if left untreated. Encouragingly, early ART largely reversed CSF metabolic imbalance in a timing-dependent manner. Treatment initiation at 3 dpi most effectively shifted the metabolome toward a healthy state, while later initiation at 5 dpi only partially restored metabolic balance. Several altered metabolites, including phosphocreatine, sorbitol, and kynurenine, may serve as candidate biomarkers of disease severity and therapeutic efficacy. These findings parallel clinical observations that early ART improves neurodevelopmental outcomes in HIV-infected infants [61, 62].

In summary, neonatal SIV infection is characterized by a short delay in brain reservoir seeding, minimal early neuropathology, and profound metabolic disruption in the CSF. Importantly, timely early treatment not only prevents the establishment of brain reservoirs but also restores metabolic homeostasis, thereby preserving CNS development. These findings underscore the urgent need for immediate therapeutic intervention in HIV-exposed neonates to maximize the chances of achieving functional cure and protecting long-term brain health.

## Data Availability

All other data and materials are available upon reasonable request from the correspondingauthors.
